# MR imaging phenotypes and features associated with pathogenic mutation to predict recurrence or metastasis in breast cancer

**DOI:** 10.1186/s12885-023-10555-5

**Published:** 2023-01-27

**Authors:** Zhenzhen Shao, Jinpu Yu, Yanan Cheng, Wenjuan Ma, Peifang Liu, Hong Lu

**Affiliations:** 1 Department of Breast Imaging, Tianjin Medical University Cancer Institute & Hospital, National Clinical Research Center for Cancer, Tianjin’s Clinical Research Center for Cancer, Key Laboratory of Breast Cancer Prevention and Therapy, Tianjin Medical University, Ministry of Education, Key Laboratory of Cancer Prevention and Therapy, Tianjin, P. R. China; 2 Cancer Molecular Diagnostics Core, Tianjin Medical University Cancer Institute & Hospital, National Clinical Research Center for Cancer, Tianjin’s Clinical Research Center for Cancer, Key Laboratory of Breast Cancer Prevention and Therapy, Tianjin Medical University, Ministry of Education, Key Laboratory of Cancer Prevention and Therapy, Tianjin, P. R. China

**Keywords:** Breast cancer, MRI phenotypes, Biologic features, Pathogenic mutation, Disease recurrence or metastasis

## Abstract

**Objectives:**

Distant metastasis remains the main cause of death in breast cancer. Breast cancer risk is strongly influenced by pathogenic mutation.This study was designed to develop a multiple-feature model using clinicopathological and imaging characteristics adding pathogenic mutations associated signs to predict recurrence or metastasis in breast cancers in high familial risk women.

**Methods:**

Genetic testing for breast-related gene mutations was performed in 54 patients with breast cancers. Breast MRI findings were retrospectively evaluated in 64 tumors of the 54 patients. The relationship between pathogenic mutation, clinicopathological and radiologic features was examined. The disease recurrence or metastasis were estimated. Multiple logistic regression analyses were performed to identify independent factors of pathogenic mutation and disease recurrence or metastasis. Based on significant factors from the regression models, a multivariate logistic regression was adopted to establish two models for predicting disease recurrence or metastasis in breast cancer using R software.

**Results:**

Of the 64 tumors in 54 patients, 17 tumors had pathogenic mutations and 47 tumors had no pathogenic mutations. The clinicopathogenic and imaging features associated with pathogenic mutation included six signs: biologic features (*p* = 0.000), nuclear grade (*p* = 0.045), breast density (*p* = 0.005), MRI lesion type (*p* = 0.000), internal enhancement pattern (*p* = 0.004), and spiculated margin (*p* = 0.049). Necrosis within the tumors was the only feature associated with increased disease recurrence or metastasis (*p* = 0.006). The developed modelIincluding clinico-pathologic and imaging factors showed good discrimination in predicting disease recurrence or metastasis. Comprehensive model II, which included parts of modelIand pathogenic mutations significantly associated signs, showed significantly more sensitivity and specificity for predicting disease recurrence or metastasis compared to Model I.

**Conclusions:**

The incorporation of pathogenic mutations associated imaging and clinicopathological parameters significantly improved the sensitivity and specificity in predicting disease recurrence or metastasis. The constructed multi-feature fusion model may guide the implementation of prophylactic treatment for breast cancers at high familial risk women.

## Key points

1. Only two MRI phenotypes –“internal enhancement patterns” and “central necrosis” were associated with disease recurrence or metastasis.
2. The MRI phenotypes associated with pathogenic mutation adding to the model based clinicopathologic and imaging factors for predicting disease recurrence or metastasis, the comprehensive model can improve the predicting performance.

## Introduction

Breast cancer is the most commonly diagnosed cancer, ranking fifth in the mortality of cancers in women in China [1]. Breast cancer risk is strongly influenced by genetic factors. The *BRCA1* and *BRCA2* are key genes associating with the development of breast cancer [2, 3]. *BRCA1* mutation carriers are more likely to have triple-negative and higher nuclear grade, which tend to have bad prognoses [4]. In addition, patients with metastasis or recurrence have also worse prognoses. Thus, many efforts to predict metastasis or recurrence in breast cancer patients, especially adding factors associated with pathogenic gene mutations have been made.

Magnetic resonance imaging (MRI) has been widely used for screening women at increased risk for breast cancer [5]. The sensitivity of MRI for detecting breast tumors ranges from 77 to 91%, which is higher than mammography (33%-40%), in women at high familial risk for breast cancer [6, 7]. Breast MRI can accurately diagnose breast cancer and predict prognosis using variable imaging features. Previous studies identified that peritumoral edema on T2-weighted images and higher degree of background parenchymal enhancement (BPE), and increased vascularity around the tumor, indicate poor prognosis in breast cancer [8–10]. Some studies have also demonstrated breast cancers with *BRCA* mutations had characteristic performances with round shape, sharp margins, and rim enhancement [11–13]. However, few studies have been reported on the relationship between pathogenic mutations and MRI features. In addition, multigene panel testing using NGS (next generation sequencing) technology could identify up to 50% more individuals with cancer susceptibility gene mutations in comparison with testing only for *BRCA1* and *BRCA2* [14]. Moreover, NGS technology has become possible to study a wider range of hereditary cancer related genes. The sequential analysis of genes has the disadvantage of being expensive, laborious and time consuming.

Therefore, the aim of this study was to retrospectively identify signs associated with pathogenic mutations in 16 genes, and furthermore to develop a multi-feature model using clinicopathological features and radiologic characteristics in addition to pathogenic mutations associated signs for predicting metastatic disease or recurrences in high familial risk women.

## Materials and methods

### Patients

This retrospective study included patients with histologically confirmed invasive ductal carcinomas and ductal carcinomas in situ from July 2014 to January 2016. Individual consent for this retrospective analysis was waived, and was approved by the institutional ethics board of Tianjin Medical University Cancer Institute and Hospital (Ek2018125). A total of 100 patients were initially included in the study according to the following inclusion criteria: (a) preoperative MR images were acquired, (b) available pathological and immune-histochemical reports, (c) available test results for gene mutation status, (d) available clinical data, including age, familial history of breast cancer, and TNM stage. Among them, 44 patients were excluded due to the following exclusion criteria: (a) patients with confirmed benign lesions (*n* = 23), (b) patients received preoperative neoadjuvant chemotherapy treatment (*n* = 11), (c) patients received local resection of lesions before MRI examinations (*n* = 10). In the end, 64 tumors of 56 patients were included, unilateral breast cancers in 48 patients, bilateral breast cancers in 6 patients, and two tumors of unilateral breast in 2 patients.

### Imaging technique

Dynamic breast MR imaging was performed using a 3.0 T magnet (GE Discovery 750) with patients in the prone position using a dedicated double-breast coil. Both breasts were imaged with axial T1-weighted sequences (TR = 622 ms,TE = 10 ms), fat-suppressed axial T2-weighted sequences (TR = 6330 ms,TE = 68 ms), and axial DWI sequences (TR = 3235 ms, TE = 64 ms) with *b* values of 0 and 1000 s/mm^2^. The contrast enhancement scan used was a sagittal T1-weighted fat-suppressed volume imaging for breast assessment (VIBRANT) sequence (TR = 6.1 ms, TE = 2.9 ms, flip angle = 15°, matrix = 256 × 128, slice thickness = 1.8 mm, phase acquisition time = 90 ~ 100 s), obtained before and five continuous times after intravenous injection of 0.1 mmol/L gadolinium chelate per kilogram body weight (Gadovist, Bayer Schering Pharma, Berlin, Germany).

### Imaging interpretation

Two radiologists (a junior and a senior radiologist with 5 and 10 years of breast MRI experience, respectively) individually read the MRI images, when the two radiologists’ results were found to be discordant, the results were decided by the senior radiologist. The inter-reader consistency was high (Kappa = 0.874, *p* < 0.001) as determined by Kappa statistics analysis.The MR imaging characteristics of tumors were described by using terminology defined by BI-RADS [15]. Breast density included dense (heterogeneously dense and extremely dense) and non-dense (entinely fatty and scattered fibroglandular density). The lesion type was classified as mass, asymmetry/distortion, and calcifications on mammography and as mass and nonmass enhancement on MRI. The shape (regular or irregular), internal enhancement pattern (heterogeneous mass-enhancement, rim mass-enhancement, or linear/segmental nonmass-enhancement), spiculated margins (yes or no), central necrosis within the tumor (yes or no), peritumoral edema (yes or no), and tumor localization (edge or central) were then evaluated on MRI. The edge was defined as the boundary between the glands and subcutaneous fat. The central was defined as the interior of the glands.

### Histologic evaluation and pathogenic mutation analysis

Histopathology, nuclear grade, tumor stage (T\N\M), and the status of immunohistochemical (IHC) staining for ER, PR, p53, Ki67, and HER2 were recorded, and positivity for ER and PR was defined by the ASCO-CAP guidelines [16]. Positive HER2 status was determined using IHC 3 + staining (more than 10% of infiltrating cancer cells showed strong and intact cell membrane staining), or amplification using fluorescence in situ hybridization. Molecular subtypes were classified into Luminal A, Luminal B, HER2 over-expression, and Triple negative. Subtypes were further classified into two groups including triple-negative (ER, PR, and HER negative) and non triple-negative. Biologic features were classified into three groups: unfavorable group (intermediate nuclear grade and ER negative/ PR negative, high nuclear grade and ER negative/ PR negative, high nuclear grade and ER negative/PR positive, high nuclear grade and ER positive/PR negative), favorable group (low nuclear grade and ER positive/ PR positive, low nuclear grade and ER negative/PR positive, low nuclear grade and ER positive/PR negative), and intermediate group (the expressions of nuclear grade and ER/PR in addition to the above unfavorable and favorable designation). Disease recurrence or metastasis was defined as recurrence of breast cancer at any site (including local, regional, or distant).

Ion Ampliseq Designer (https://ampliseq.com/ Browse. action) was used to design the multiplex PCR amplification primers of the exon regions of 16 inherited breast cancer related genes (*BRCA1*, *BRCA2*, *CHEK2*, *PALB2*, *BRIP1*, *TP53*, *PTEN*, *STK11*, *CDH1*, *ATM*, *BARD1*, *MLH1*, *MRE11A*, *MSH2*, *MSH6*, and *BAP1*) with a coverage rate of 99.8%. Sanger sequencing was used to detect areas that could not be covered by the Ampliseq panel. According to the kit instructions, genomic DNA was extracted from the peripheral blood of breast cancer patients, and the DNA was amplified by multiplex PCR (Polymerase Chain Reaction). The amplified products were connected, purified, oil-in-water reaction and enrichment of positive templates. Samples were sequenced in a Ion Proton using GPM 314 chip. After sequencing, both Coverage Analysis and Variant Caller datasets were downloaded, and IGV software was used to analyze the results and determine whether there were pathogenic mutation genes in the 16 genes.

### Statistical analysis

The chi-square and Fisher exact tests were used to compare proportions between the two study groups (pathogenic mutation vs. no pathogenic mutation and disease recurrence or metastasis vs. no disease recurrence or metastasis) by using SPSS software (version 20.0). Multiple logistic regression analyses were performed to identify independent factors that can be used to predict pathogenic mutation and disease recurrence or metastasis. Based on the above significant factors, a multivariate logistic regression was adopted to establish two models for predicting disease recurrence or metastasis in breast cancer by using R software (version 6.1, R Foundation for Statistical Computing, Vienna, Austria). Two-size *P* < 0.05 indicated a significant difference. Model I included clinicopathologic features and imaging variables, and Model II included clinicopathologic features and imaging variables in addition to pathogenic mutations associated parameters. The performance was evaluated by the area under the receiver operating characteristic (ROC) curve, accuracy, sensitivity, and specificity. The difference in the area under the curve (AUC) between Model I and Model II was analyzed by Delong’s test.

## Results

### Gene mutations status

**Fig. 1:**
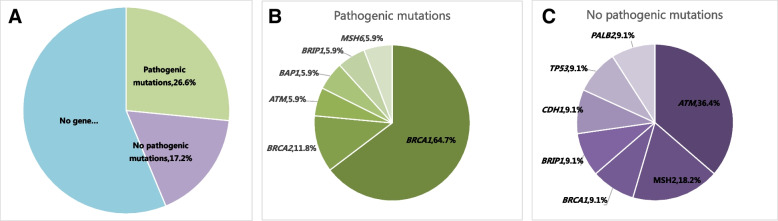
Distribution of the 16 susceptibility mutations in 64 breast carcinomas. **A** distribution of gene mutations; **B** distribution of pathogenic mutations; **C** distribution of no pathogenic mutations. Genes not shown (*PTEN, STK11, CHEK2, BARD1, MLH1, MRE11A*) are those without identified mutations in the study cohort

All 64 tumors in 56 patients (mean age, 43.5 years; age range, 26–72 years) were included. All patients had a family history of breast cancer. Of 64 tumors, gene mutations were detected in 28 tumors (43.8%) (Fig. 1), pathogenic mutations were identified in 17(26.6%) of 64 tumors, with 11 (17.2%) carrying *BRCA1* mutations, 2 (3.1%) carrying *BRCA2* mutations, and 4 (6.3%) with pathogenic mutations in other genes including *ATM* (*n* = 1), *BAP1* (*n* = 1), *BRIP1* (*n* = 1), and *MSH6* (*n* = 1), respectively. Table 1 summarizes the 17 identified pathogenic mutations, which contained 10 (58.8%) frameshift, 4 (23.5%) nonsense, and 3 (17.6%) splice-site variants in 17 breast cancers. Non-pathogenic mutations were identified 11(17.2%) in 64 tumors, including 4 carrying *ATM* mutations, 2 carrying *MSH2* mutations, 1 carrying *BRIP1* mutation, 1 carrying *BRCA1* mutation,1 carrying *PALB2* mutation, 1 carrying *CDH1* mutation, and 1 carrying *TP53* mutation. Table 2 summarizes the 11 identified non- pathogenic mutations, which all were missense in 11 breast cancers. The other 36 tumors had no gene mutations in the 16 genes.

**Table 1:** Pathogenic gene mutations in 15 patients with 17 cancers

Gene	Mutation	Age at diagnosis	Family history	Mutation type
*BRCA1*	c.3359_3363delTTAAT	48	Sister	frameshift
	c.5470_5477delATTGGGCA	30	Aunt	frameshift
	c.5136G > A^a^	29	Mother	nonsense
	c.5153-1G > A^a^	47	Sister	splice-site variants
	chr17:41246509delG	41	Mother	frameshift
	chr17:41226484insC	44	Sister	frameshift
	c.5470_5477delATTGGGCA	53	Aunt	frameshift
	chr17:41226455delG	49	Mother	frameshift
	c.2138C > G	34	Mother, Aunt	nonsense
*BRCA2*	chr13:32913702DelTACT	51	Mother	frameshift
	chr13:32914936InsTA	41	Sister	frameshift
*BAP1*	chr3:52440911InsC	26	Mother	frameshift
*BRIP1*	chr17:59793425C > A	41	Sister	splice-site variants
*MSH6*	chr2:48010546delC	49	Mother	frameshift
*ATM*	c.2414C > T	32	Aunt	nonsense

^a^Indicates that the patient had two tumors

**Table 2:** No pathogenic mutations in 11 patients with 11 cancers

Gene	Mutation	Age at diagnosis	Family history	Mutation type
*BRIP1*	chr17:59938867C > A	75	Sister	missense
*BRCA1*	c.154C > T	46	Sister	missense
*PALB2*	c.3296C > T	43	Sister	missense
*ATM*	c.8716G > C	47	Sister	missense
	c.275A > C	49	Mother	missense
	c.125A > G	43	Aunt	missense
	c.8495G > A	38	Mother	missense
*CDH1*	c.1888C > G	45	Sister	missense
*MSH2*	c.970C > T	42	Mother	missense
	c.14C > A	63	Mother	missense
*TP53*	c.733G > A	32	Mother	missense

No pathogenic mutations included non-pathogenic mutations in gene mutations

### Clinicopathological and radiologic features associated with pathogenic mutations

The associations between clinico-pathological factors and pathogenic mutation status are presented in Table 3. The women younger than 40-year-old with breast cancer had more detected pathogenic gene mutations than the women older than 40-year-old (*p* = 0.039). The pathogenic gene mutations subgroup had a significantly higher number of cancers with high nuclear grade (6 of 17 [35.3%], *p* = 0.017), ER negative (7 of 17 [41.2%], *p* = 0.004), and PR negative (7 of 17 [41.2%], *p* = 0.007). No significant association was observed between histopathology, T stage, N stage, HER2 overexpression, p53 status and the pathogenic gene mutation. A near significant difference in Ki67 status (*p* = 0.057) between the pathogenic mutations and no pathogenic mutations groups. Pathogenic mutations were more likely to be the triple-negative phenotype (9 of 17 [52.9%]) than another subtype (3 of 47 [6.4%]) (*p* = 0.000). Significant pathogenic mutation rates were observed in unfavorable biological behavior cancers (10 of 17 [58.8%], *p* = 0.000). Breast cancers carrying pathogenic mutations were more likely to be associated with recurrence or metastasis (5 of 17 [29.4%] and 3 of 47 [6.4%]) (*p* = 0.014).

**Table 3:** Clinicopathological characteristics of breast cancers with and without pathogenic mutations

Variable	Pathogenic *N* = 17	No Pathogenic *N* = 47	No. of all	*P* value
**Age**				
< 40 years	7 (41.2%)	7 (14.9%)	14 (21.9%)	**0.039**
≥ 40 years	10 (58.8%)	40 (85.1%)	50 (78.1%)	
**Histopathological type**				
Ductal carcinoma in situ	1 (5.9%)	7 (14.9%)	8(12.5%)	0.434
Invasive ductal carcinoma	16 (94.1%)	40 (85.1%)	56 (87.5%)	
**Tumor size**				
T1	8 (47.1)	15 (31.9)	23 (35.9)	0.534
T2	7(41.2)	26 (55.3)	33 (51.6)	
T3	2 (11.8)	6 (12.8)	8 (12.5)	
**Node metastasis**				
N-	10 (58.8)	34 (72.3)	44 (68.8)	0.365
N +	7 (41.2)	13 (27.7)	20 (31.2)	
**Recurrence or Metastasis**				
Yes	5 (29.4)	3 (6.4)	8 (12.5)	**0.014**
No	12 (70.6)	44 (93.6)	56 (87.5)	
**Biologic feature**				
unfavorable	10(58.8)	6(12.8)	16(25.0)	**0.000**
Intermediate/ favorable	7(41.2)	41(87.2)	48(75.0)	
**Nuclear grade**				
Low/Intermediate	11 (64.7)	43(91.5)	54 (84.4)	**0.017**
high	6 (35.3)	4 (8.5)	10(15.6)	
**ER**				
ER-	10 (58.8)	9(19.1)	19 (29.7)	**0.004**
ER +	7 (41.2)	38 (80.9)	45 (70.3)	
**PR**				
PR-	10(58.8)	10(21.3)	20(31.2)	**0.007**
PR +	7(41.2)	37 (78.7)	44 (68.8)	
**HER2**				
HER2-	13 (76.5)	29 (61.7)	42 (65.6)	0.375
HER2 +	4 (23.5)	18 (38.3)	22 (34.4)	
**p53**				
p53-	5 (29.4)	24 (51.1)	29 (45.3)	0.160
p53 +	12 (70.6)	23 (48.9)	35 (54.7)	
**Ki67**				
Ki67-	1 (5.9)	13 (27.7)	14 (21.9)	**0.057**
Ki67 +	16 (94.1)	34 (72.3)	50 (78.1)	
**Molecular subtype**				
Luminal A	3 (17.6)	15 (31.9)	18 (28.1)	
Luminal B	5 (29.5)	24 (51.1)	29 (45.3)	**0.000**
HER2 over-expression	0 (0.0)	5 (10.6)	5 (7.8)	
Triple negative	9 (52.9)	3 (6.4)	12 (18.8)	
**Triple negative**				
No	8 (47.1)	44 (93.6)	52 (81.2)	**0.000**
Yes	9 (52.9)	3 (6.4)	12 (18.8)	

The “No Pathogenic” included the non-pathogenic mutations in gene mutations and no gene mutations

The mammographic and contrast-enhanced MR characteristics of the tumors in each genetic subgroup are presented in Table 4. On mammography, dense breast tissue in patients with pathogenic mutations (16 of 17 [94.1%]) were significantly denser than those in patients with no pathogenic mutations (30 of 47 [63.8%]) (*p* = 0.025). Mammographic lesion features were not significantly associated with the pathogenic mutation status (*p* = 0.722). On MRI scan, cancers were identified as masses type in all 17 lesions (100%) with pathogenic mutation, and as masses type in 37 lesions (78.7%) and as nonmass enhancement in 10 lesions (21.3%) with no pathogenic mutations (*p* = 0.038). We found differences in internal enhancement patterns (*p* = 0.000), spiculated margins (*p* = 0.005), or necrosis within tumor (*p* = 0.009) when comparing the pathogenic and the non-pathogenic mutation subtypes. Breast cancers carrying pathogenic mutations showed ring mass enhancement more frequently with non-spiculated margins and necrosis within the tumor. No significant associations were found between pathogenic mutation status with the shape (*p* = 0.253), edema around tumor (*p* = 0.847), or the location of the cancer (*p* = 0.623).

**Table 4:** Imaging features of breast cancers with and without pathogenic mutations

Variable	Pathogenic *N* = 17	No Pathogenic *N* = 47	No. of all	*P* value
**MG**				
**Breast density**				
Non-dense	1(5.9)	17(36.2)	18(28.1)	**0.025**
Dence	16(94.1)	30(63.8)	46(71.9)	
**Lesion type**				
Calcification	3 (17.6)	5 (10.6)	8 (12.5)	0.722
Mass	7 (41.2)	19 (40.4)	26 (40.6)	
Asymmetry/ Distortion	7(41.2)	23 (48.9)	30 (46.9)	
**MRI**				
**Lesion type**				
Mass	17(100.0)	37 (78.7)	54 (84.4)	**0.038**
Nonmass	0(0.0)	10 (21.3)	10 (15.6)	
**Internal enhancement pattern**				
Heterogeneous mass-enhancement	4 (23.5)	30 (63.8)	34 (53.2)	**0.000**
Rim mass-enhancement	13 (76.5)	7 (14.9)	20 (31.2)	
Line/segmental nonmass-enhancement	0 (0.0%)	10 (21.3)	10 (15.6)	
**Shape**				
Regular	6 (35.3)	10 (21.3)	16 (25.0)	0.253
Irregular	11 (64.7)	37 (78.7)	48 (75.0)	
**Spiculated margin**				
Yes	0 (0.0)	16 (34.0)	16 (25.0)	**0.005**
No	17 (100.0)	31 (66.0)	48 (75.0)	
**Central necrosis**				
Yes	6 (35.3)	4 (8.5)	10 (15.6)	**0.009**
No	11 (64.7)	43 (91.5)	54 (84.4)	
**Peritumoral edema**				
Yes	4 (23.5)	10 (21.3)	14 (21.9)	0.847
No	13 (76.5)	37 (78.7)	50 (78.1)	
**Tumor localization**				
Edge	14 (82.4)	36 (76.6)	50 (78.1)	0.623
Central	3 (17.6)	11 (23.4)	11 (21.9)	

### Clinico-pathological and radiologic features associated with disease recurrence or metastasis

The 5-year rate of disease recurrence or metastasis was 12.5% (8/64) for all patients, 29.4% (5/17) for the pathogenic mutation subgroup and 6.4% (3/47) for the no pathogenic mutation subgroup, which was significant difference (*p* = 0.014). Tumors with five features including high nuclear grade (4 of 8 [50.0%], *p* = 0.004), ER negative (5 of 8 [62.5%], *p* = 0.030), PR negative (5 of 8 [62.5%], *p* = 0.041), p53 positive (7 of 8 [87.5%], *p* = 0.046) and triple negative subtype (4 of 8 [50.0%], *p* = 0.015) had more disease recurrence or metastasis during the 5 years after surgery (Table 5). Only two radiologic features – “internal enhancement patterns” (*p* = 0.045) and “central necrosis” (*p* = 0.000) were associated with disease recurrence or metastasis (Table 6). Breast cancers with ring mass enhancement and central necrosis were more likely to be recurrence or metastasis.

**Table 5:** Clinicopathological characteristics of breast cancers with and without recurrence or metastasis

Variable	Recurrence or Metastasis *N* = 8	No Recurrence or Metastasis *N* = 56	No. of all	*P* value
**Age**				
< 40 years	3 (37.5)	11 (19.6)	14 (21.9)	0.253
≥ 40 years	5 (62.5)	45 (80.4)	50 (78.1)	
**Histopathological type**				
Ductal carcinoma in situ	1 (12.5)	7 (87.5)	8(12.5)	1.000
Invasive ductal carcinoma	7 (12.5)	49 (87.5)	56 (87.5)	
**Tumor size**				
T1	2 (25.0)	21 (37.5)	23 (35.9)	0.073
T2	3 (37.5)	30 (53.6)	33 (51.6)	
T3	3 (37.5)	5 (8.9)	8 (12.5)	
**Node metastatic**				
N-	5 (62.5)	39 (69.6)	44 (68.8)	0.683
N +	3 (37.5)	17 (30.4)	20 (31.2)	
**Biologic feature**				
unfavorable	4(50.0)	12(21.4)	16(25.0)	0.081
Intermediate/ favorable	4(50.0)	44(78.6)	48(75.0)	
**Nuclear grade**				
Low/Intermediate	4 (50.0)	50(89.3)	54 (84.4)	**0.004**
high	4 (50.0)	6 (10.7)	10(15.6)	
**ER**				
ER-	5 (62.5)	14 (25.0)	19 (29.7)	**0.030**
ER +	3 (37.5)	42 (75.0)	45 (70.3)	
**PR**				
PR-	5 (62.5)	15 (26.8)	20 (31.2)	**0.041**
PR +	3 (37.5)	41 (73.2)	44 (68.8)	
**HER2**				
HER2-	7 (87.5)	35 (62.5)	42 (65.6)	0.164
HER2 +	1 (12.5)	21 (37.5)	22 (34.4)	
**p53**				
p53-	1 (12.5)	28 (50.0)	29 (45.3)	**0.046**
p53 +	7 (87.5)	28 (50.0)	35 (54.7)	
**Ki67**				
Ki67-	0 (0.0)	14 (25.0)	14 (21.9)	0.110
Ki67 +	8(100.0)	42 (75.0)	50 (78.1)	
**Molecular substype**				
Luminal A	0 (0.0)	18 (32.1)	18 (28.1)	0.052
Luminal B	3 (37.5)	26 (46.4)	29 (45.3)	
HER2 over-expression	1 (12.5)	4 (7.2)	5 (7.8)	
Triple negative	4 (50.0)	8 (14.3)	12 (18.8)	
**Triple negative**				
No	4 (50.0)	48 (85.7)	52 (81.2)	**0.015**
Yes	4 (50.0)	8 (14.3)	12 (18.8)

**Table 6:** Imaging features of breast cancers with and without recurrence or metastasis

Variable	Recurrence or Metastasis *N* = 8	No Recurrence or Metastasis *N* = 56	No. of all	*P* value
**MG**				
**Breast density**				
Non-dense	1 (12.5)	17 (30.4)	18(28.1)	0.293
Dense	7 (87.5)	39 (69.6)	46(71.9)	
**Lesion type**				
Calcification	1 (12.5)	7 (12.5)	8 (12.5)	0.831
Mass	3 (37.5)	22 (39.3)	26 (40.6)	
Asymmetry/ Distortion	4 (50.0)	27 (48.2)	30 (46.9)	
**MRI**				
**Lesion type**				
Mass	6(75.0)	48 (85.7)	54 (84.4)	0.435
Nonmass	2(25.0)	8 (14.3)	10 (15.6)	
**Internal enhancement pattern**				
Heterogeneous mass-enhancement	1 (12.5)	33 (58.9)	34 (53.1)	**0.045**
Rim mass-enhancement	5 (62.5)	15 (26.8)	20 (31.2)	
Line/segmental nonmass-enhancement	2 (25.0)	8 (14.3)	10 (15.6)	
**Shape**				
Regular	4 (50.0)	12 (21.4)	16 (25.0)	0.081
Irregular	4 (50.0)	44 (78.6)	48 (75.0)	
**Spiculated margin**				
Yes	0 (0.0)	16 (28.6)	16 (25.0)	0.081
No	8 (100.0)	40 (71.4)	48 (75.0)	
**Central necrosis**				
Yes	6(75.0)	4 (7.1)	10 (15.6)	**0.000**
No	2(25.0)	52 (92.9)	54 (84.4)	
**Peritumoral edema**				
Yes	3 (37.5)	11 (19.6)	14 (21.9)	0.253
No	5 (62.5)	45 (80.4)	50 (78.1)	
**Tumor localization**				
Edge	6 (75.0)	44 (78.6)	50 (78.1)	0.819
Central	2 (25.0)	12 (21.4)	14 (21.9)	

### Multivariable analyses of predicting pathogenic mutations and disease recurrence or metastasis and ROC analyses

**Fig. 2:**
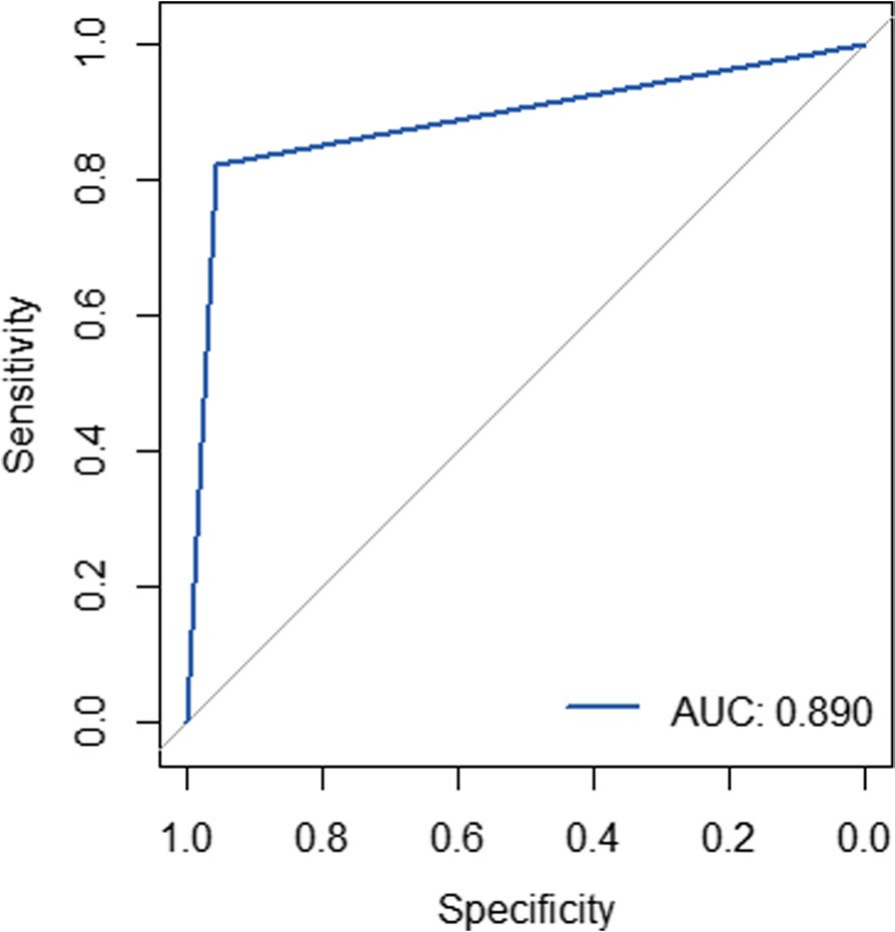
Receiver operating characteristic curves used to predict pathogenic mutation with clinicopathologic and imaging features

Six features including biologic feature, nuclear grade, breast density, MRI lesion type, internal enhancement pattern and non-spiculated margin were shown to be significantly independent prognostic factors predicting pathogenic mutations by multivariable logisitic regression. Logistic regression was performed using the above significant clinicopathological and imaging features obtained by univariate analysis in Table 7. The AUC was 0.890 (95%CI: 0.793–0.988) (Fig. 2), the sensitivity and specificity were 0.937 and 0.875, respectively.

**Table 7:** Multiple logistic regression analysis predicting pathogenic mutation on breast cancers

Variable	Odds ratio	95%CI	*P* value
**Age**			
< 40 years	Reference	NA	0.418
≥ 40 years	0.914	0.736–1.134	
**Recurrence or Metastasis**			
No	Reference	NA	**0.039**
Yes	1.402	1.024–1.916	
**Biologic feature**			
unfavorable	Reference	NA	**0.000**
Intermediate/ favorable	0.409	0.259–0.647	
**Nuclear grade**			
Low/Intermediate	Reference	NA	**0.045**
high	0.736	0.551–0.986	
**ER**			
ER +	Reference	NA	0.416
ER-	1.368	0.646–2.896	
**PR**			
PR +	Reference	NA	0.304
PR-	1.399	0.742–2.642	
**Molecular substype**			
Non-Triple negative	Reference	NA	0.634
Triple negative	1.099	0.721–1.676	
**Breast density**			
Non-dense	Reference	NA	**0.005**
Dense	1.315	1.093–1.582	
**MRI Lesion type**			
Nonmass	Reference	NA	**0.000**
Mass	2.705	1.739–4.207	
**Internal enhancement pattern**			
Other enhancement types	Reference	NA	**0.004**
Rim mass-enhancement	1.404	1.121–1.758	
**Spiculated margin**			
Yes	Reference	NA	**0.049**
No	0.803	0.649–0.994	
**Central necrosis**			
No	Reference	NA	0.388
Yes	0.875	0.649–1.181

**Fig. 3:**
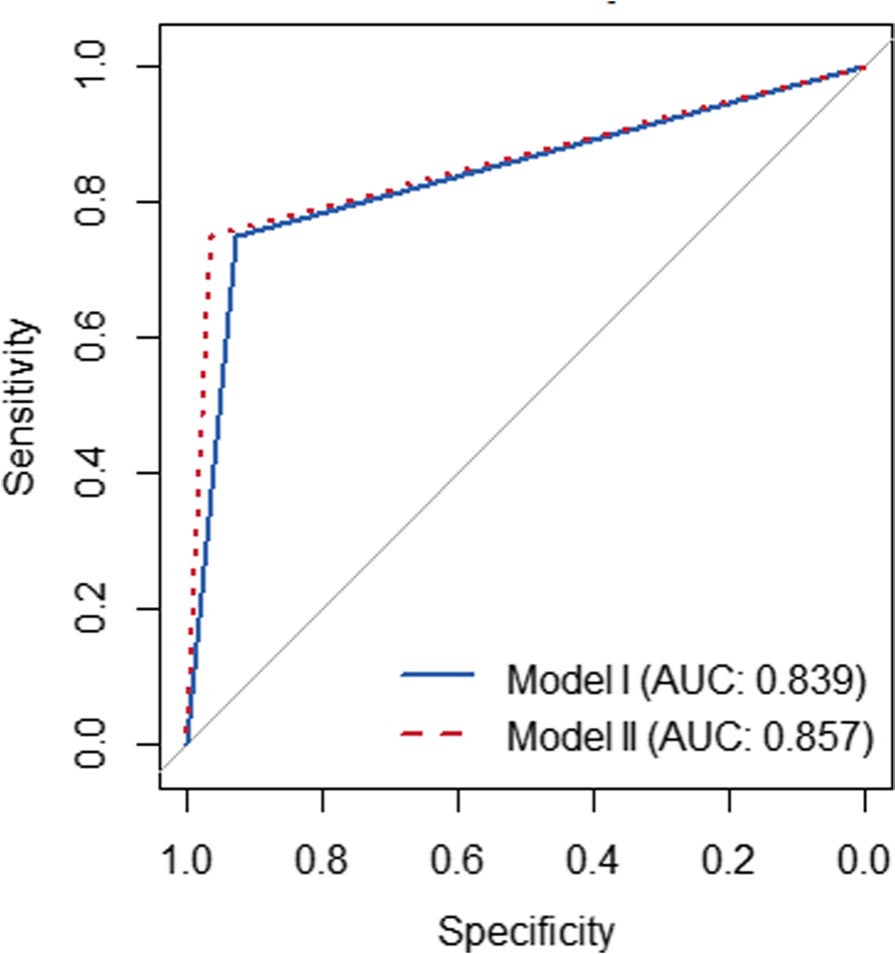
Receiver operating characteristic curves used to predict recurrence or metastasis with clinicopathologic and imaging features alone (Model I) and combined with signs significantly associated with pathogenic mutation (Model II)

Seven clinicopathologic and imaging features including nuclear grade, ER, PR, p53, molecular subtype, internal enhancement pattern, and central necrosis were analyzed by using multivariable logistic regression and reported in Table 8. One important and significant prognostic factor central necrosis within the tumor, was used to develop the Model I for predicting disease recurrence or metastasis. The AUC of Model I was 0.839 (95%CI: 0.675–1.000) (Fig. 3), the sensitivity and specificity were 0.963 and 0.600, respectively. When pathogenic mutations status was added to Model I, the AUC, sensitivity, and the specificity were consistent to the Model I prediction of disease recurrence or metastasis.

**Table 8:** Multiple logistic regression analysis predicting recurrence or metastasis on breast cancers

Variable	Odds ratio	95%CI	*P* value
**Nuclear grade**			
Low/Intermediate	Reference	NA	0.056
high	1.242	0.998–1.544	
**ER**			
ER +	Reference	NA	0.888
ER-	1.042	0.590–1.839	
**PR**			
PR +	Reference	NA	0.920
PR-	0.973	0.573–1.653	
**p53**			
p53-	Reference	NA	0.348
p53 +	1.069	0.930–1.230	
**Molecular subtype**			
Non-Triple negative	Reference	NA	0.498
Triple negative	0.909	0.691–1.196	
**Internal enhancement pattern**			
Other enhancement types	Reference	NA	0.439
Rim mass-enhancement	1.037	0.946–1.138	
**Central necrosis**			
No	Reference	NA	**0.000**
Yes	1.671	1.357–2.056

The multiple-feature model (Model II) using clinicopathological, imaging characteristics, and pathogenic mutations associated signs was developed to predict recurrence or metastasis. The AUC of Model II was 0.857 (95%CI: 0.695–1.000) (Fig. 3), the sensitivity and specificity was 0.964 and 0.750, respectively. When clinicopathologic and imaging features significantly associated with pathogenic mutations were added, the AUC increased from 0.839 to 0.857, but did not make a significant difference (*P* = 0.153), and the specificity increased from 0.600 to 0.750 (Fig. 3).

## Discussion

In our study, there was a 26.6% carrier rate of pathogenic mutations in the 64 breast cancers. These cancers had a 20.3% (13/64) in high-penetrance and 6.3% (4/64) moderate/low- penetrance of the pathogenic mutation genes and the prevalence of *BRCA* mutation was 20.3% (13/64). Tsaousis et al. [17] showed that the pathogenic mutation frequencies of high-risk and moderate-risk genes was 16.5% and 6.2% respectively. Wang et al. [18] detected a 13.5% carrier rate of pathogenic germline mutation in the 20 genes, but a higher carrier rate (26.6%) of pathogenic mutations was observed in this study. The high rate may be a result that our enrolled women had high risk with a family history of breast cancer.

Our results indicated that pathogenic mutations were associated with women younger than 40 years old, high nuclear grade, triple-negative subtypes, and unfavorable biological behavior, as well as dense breasts on mammography. We also found that breast cancers with pathogenic mutations exhibited ring mass enhancement with non-spiculated margins on MRI. In this study, the pathogenic mutation subgroup and the non-pathogenic mutation subgroup were compared and analyzed. Compared to other similar studies focusing on the *BRCA* mutations, 76.5% (13/17) contained the *BRCA* mutations in the pathogenic mutations subgroup of this study. Some studies had shown that a significant number of lesions described as rounded, with sharp margins, and with ring-enhancement was found to be higher in the BRCA mutations [11, 13]. Yip et al. [19] found that in the Asian population there is a significant association between triple negative and *BRCA1* but not *BRCA2* status and a trend toward a higher percentage of grade 3 cancers in the *BRCA1* carriers but not in the *BRCA2* carriers. These above results were consistent with the characteristics observed in the current study. In this study, we also found breast cancers with central necrosis were more frequently associated with pathogenic mutations, disease recurrence or metastatic disease. The feature of rim-enhancement with central necrosis is associated with insufficient microvessel growth, which can be an indicator for the growth rate of tumors. Jimenez and coworkers have described centrally necrotizing carcinomas to have an accelerated clinical course and early systemic metastasis [20]. An accelerated growth rate can be associated with a high mitotic activity index (MAI) [11, 21]. Several authors have implied that, due to the rapid growth rate of tumors in gene mutation carriers, the risk of recurrence or metastasis could be high, so the screening frequency should be increased [22, 23].

**Fig. 4:**
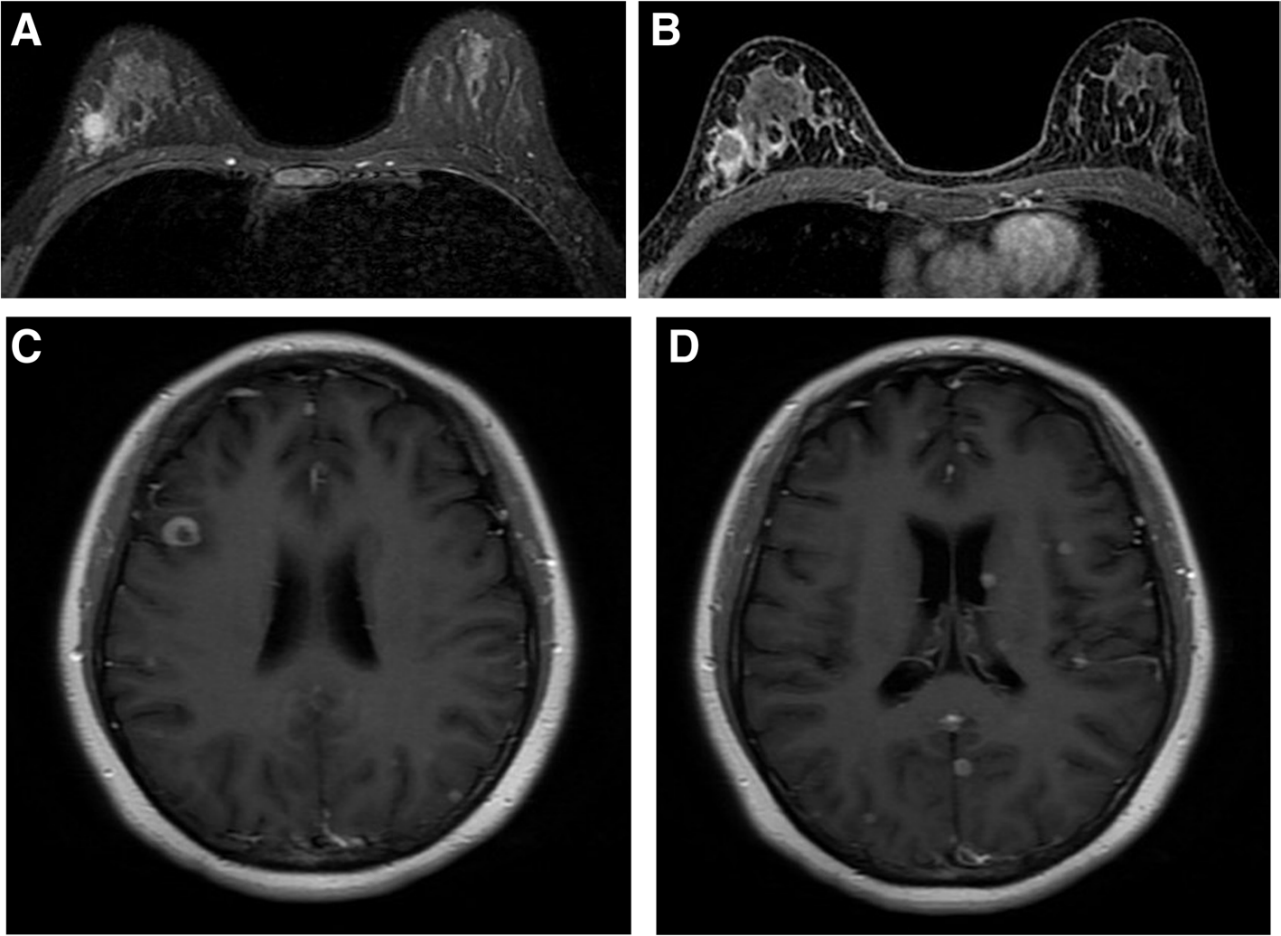
A 29-year-old woman with *BRCA1* mutation. **A** An axial fat-suppressed T2-weighted magnetic resonance image demonstrated central necrosis. **B** An axial contrast-enhanced T1-weighted magnetic resonance image demonstrated rim enhancement. **C** and **D** This patient experienced brain metastasis after 5 years from the date of surgery. Axial contrast-enhanced T1-weighted images demonstrated multiple lesions showing rim enhancement

In this study, the 5-year rate of disease recurrence or metastasis was 12.5% (8/64) for all patients, 29.4% (5/17) for the pathogenic mutation subgroup, and 6.4% (3/47) for the no pathogenic mutation subgroup. Wang et al. [18] found that the rate of disease-free survival at 5 years was 73.3% among *BRCA* mutation carriers, as compared with 91.1% among non-carriers. Previous studies demonstrated that some factors including tumour size, multiple masses, fat-saturated T_2_WI signal, were confirmed to be associated with distant metastasis [24, 25]. Some independent clinicopathologic factors were previously confirmed to be associated with distant metastasis. Age, T stage, N stage, lymphovascular invasion, and hormone receptor status were associated with bone metastasis in breast cancer [26]. One study found that sex, histological type, N stage, grade, age, ER status, PR status can predict liver metastasis [27]. Lee et al. [28] showed that increased ipsilateral vascularity and higher positive skewness of texture analysis were independently associated with disease recurrence, whereas rim enhancement showed no association with disease recurrence. In our study, central necrosis on MRI was the only factor associated with disease recurrence or metastasis, and the 5-year rate of disease-free survival was 50% (5/10) in the breast cancers with central necrosis, and 96.3% (52/54) in the breast cancers without central necrosis. Although previous studies have found that many factors affecting the prognosis, including tumor size, age, and N stage, are not statistically different because of the small sample size in this study, further studies will require further analyzsis with a larger sample size. The comprehensive multi-feature model using clinicopathological and imaging characteristics with pathogenic mutations associated signs for predicting recurrence or metastasis improved the prediction efficiency. Spanberger et al. [29] found that the extent of peritumoral edema on the preoperative T2-weighted conventional MR scan was related to the degree of angiogenesis, brain invasiveness, and overall survival. However, peritumoral edema is not a significant factor for predicting metastasis in this study, and a possible reason may be that there were brain metastases in only two patients (Fig. 4). These factors need to be further analyzed with a larger sample size. Hence, using MRI as a screening tool enables the detection of breast cancer with pathogenic mutations in women with familial risk of breast cancer.

Our study had limitations. The sample size is too small to reliably analyze the performance difference regarding the clinical and pathological characteristics. Hence, these findings should be confirmed by larger trials. Also, in this study all enrolled women had a family history of breast cancer and were analyzed not only according to *BRCA* mutation status but also according to sixteen breast cancer related genes so to further understand these results in relation to the entire population. Therefore, future studies may need broader inclusion criteria and enroll sporadic breast cancer cases from the general population. In addition, all enrolled lesions were carcinomas and the enhancement kinetic curves of all lesions were of the washout type, therefore the kinetic curve was not included in our analysis. Finally, as the multigene panel testing is becoming widely adopted, studies should develop evidence-based practice guidelines.

In summary, clinicopathologic and imaging features of breast cancers can be effective in predicting pathogenic mutations and disease recurrence or metastasis. The multi-feature model using clinicopathological, imaging characteristics, and adding pathogenic mutations associated signs enable more effective discrimination of disease recurrence or metastasis than use of clinicopathologic and imaging variables alone in breast cancers at high familial risk women.

## Data Availability

The datasets used and/or analyzed during the current study available from the corresponding author on reasonable request.

## Abbreviations

MRI: Magnetic resonance imaging
BPE: Background parenchymal enhancement
NGS: Next generation sequencing
TR: Time of repeatation
TE: Time of echo
DWI: Diffusion weighted imaging
VIBRANT: Volume imaging for breast assessment
BI-RADS: Breast imaging reporting and data system
IHC: Immunohistochemical
ER: Estrogen receptor
PR: Progesterone receptor
HER2: Human epidermal growth factor receptor-2
ASCO-CAP: American Society of Clinical Oncology-College of American Pathologists
PCR: Polymerase chain reaction
GPM: Global proteome machine
IGV: Integrative genomics viewer
ROC: Receiver operating characteristic
AUC: Area under the curve
MAI: Mitotic activity index

## References

[1] Lei S, Zheng R, Zhang S (2021). Breast cancer incidence and mortality in women in China: temporal trends and projections to 2030. Cancer Biol Med.

[2] Miki Y, Swensen J, Shattuck-Eidens D (1994). A strong candidate for the breast and ovarian cancer susceptibility gene BRCA1. Science.

[3] Wooster R, Bignell G, Lancaster J (1995). Identification of the breast cancer susceptibility gene BRCA2. Nature.

[4] Atchley DP, Albarracin CT, Lopez A (2008). Clinical and pathologic characteristics of patients with BRCA-positive and BRCA-negative breast cancer. J Clin Oncol.

[5] Kuhl C, Weigel S, Schrading S (2010). Prospective multicenter cohort study to refine management recommendations for women at elevated familial risk of breast cancer: the EVA trial. J Clin Oncol.

[6] Kriege M, Brekelmans CT, Boetes C (2004). Efficacy of MRI and mammography for breast cancer screening in women with a familial or genetic predisposition. N Engl J Med.

[7] Leach MO, Boggis CR, Dixon AK (2005). Screening with magnetic resonance imaging and mammography of a UK population at high familial risk of breast cancer: a prospective multicentre cohort study (MARIBS). Lancet.

[8] Bae MS, Shin SU, Ryu HS (2016). Pretreatment MR imaging features of triple-negative breast cancer: association with response to neoadjuvant chemotherapy and recurrence-free survival. Radiology.

[9] Hyejin C, Kim HJ, Kim TH (2018). Invasive breast cancer: prognostic value of peritumoral edema identified at preoperative MR imaging. Radiology.

[10] Lim Y, Ko ES, Han B (2017). Background parenchymal enhancement on breast MRI: association with recurrence-free survival in patients with newly diagnosed invasive breast cancer. Breast Cancer Res Treat.

[11] Veltman J, Mann R, Kok T (2008). Breast tumor characteristics of BRCA1 and BRCA2 gene mutation carriers on MRI. Eur Radiol.

[12] Noh JM, Han BK, Choi DH (2013). Association between BRCA mutation status, pathological findings, and magnetic resonance imaging features in patients with breast cancer at risk for the mutation. J Breast Cancer.

[13] Ha SM, Chae EY, Cha JH (2017). Association of BRCA mutation types, imaging features, and pathologic findings in patients with breast cancer with BRCA1 and BRCA2 mutations. AJR Am J Roentgenol.

[14] Desmond A, Kurian AW, Gabree M (2015). Clinical actionability of multigene panel testing for hereditary breast and ovarian cancer risk assessment. JAMA Oncol.

[15] American College of Radiology. ACR BI-RADS: magnetic resonance imaging. In: ACR Breast Imaging Reporting and Data System (BI-RADS) breast imaging atlas. American College of Radiology. Reston. 2013.

[16] Hammond ME, Hayes DF, Dowsett M (2010). American Society of Clinical Oncology/College of American Pathologists guideline recommendations for immunohistochemical testing of estrogen and progesterone receptors in breast cancer. Clin Oncol.

[17] Tsaousis GN, Papadopoulou E, Apessos A (2019). Analysis of hereditary cancer syndromes by using a panel of genes: novel and multiple pathogenic mutations. BMC Cancer.

[18] Wang YA (2018). Germline breast cancer susceptibility gene mutations and breast cancer outcomes. BMC Cancer.

[19] Yip CH, Taib NA, Choo WY (2009). Clinical and pathologic differences between BRCA1-, BRCA2-, and non-BRCA-associated breast cancers in a multiracial developing country. World J Surg.

[20] Jimenez RE, Wallis T, Visscher DW (2001). Centrally necrotizing carcinomas of the breast: a distinct histologic subtype with aggressive clinical behavior. Am J Surg Pathol.

[21] Tilanus-Linthorst M, Verhoog L, Obdeijn IM (2002). A BRCA1/2 mutation, high breast density and prominent pushing margins of a tumor independently contribute to a frequent false-negative mammography. Int J Cancer.

[22] Kaas R, Kroger R, Hendriks JH (2004). The significance of circumscribed malignant mammographic masses in the surveillance of BRCA 1/2 gene mutation carriers. Eur Radiol.

[23] Komenaka IK, Ditkoff BA, Joseph KA (2004). The development of interval breast malignancies in patients with BRCA mutations. Cancer.

[24] Ma W, Wang X, Xu Q (2020). Distant metastasis prediction via a multi-feature fusion model in breast cancer. Aging (Albany NY).

[25] Noda S, Onoda N, Asano Y (2015). T-stage and positive sentinel nodes ratio are the useful factors to predict non-sentinel node metastasis in breast cancer patients with macro-metastasis in the sentinel node. Int J Surg.

[26] Delpech Y, Bashour SI, Lousquy R (2015). Clinical nomogram to predict bone-only metastasis in patients with early breast carcinoma. Br J Cancer.

[27] Lin Z, Yan S, Zhang J, Pan Q (2018). A nomogram for distinction and potential prediction of liver metastasis in breast cancer patients. J Cancer.

[28] Lee J, Kim SH, Kang BJ (2020). Prognostic factors of disease recurrence in breast cancer using quantitative and qualitative magnetic resonance imaging (MRI) parameters. Sci Rep.

[29] Spanberger T, Berghoff AS, Dinhof C (2013). Extent of peritumoral brain edema correlates with prognosis, tumoral growth pattern, HIF1a expression and angiogenic activity in patients with single brain metastases. Clin Exp Metastasis.

